# Hydroxytyrosol Plays Antiatherosclerotic Effects through Regulating Lipid Metabolism via Inhibiting the p38 Signal Pathway

**DOI:** 10.1155/2020/5036572

**Published:** 2020-06-22

**Authors:** Xinxin Zhang, Yating Qin, Xiaoning Wan, Hao Liu, Chao Iv, Weibin Ruan, Li Lu, Lin He, Xiaomei Guo

**Affiliations:** ^1^Department of Cardiology, Tongji Hospital, Tongji Medical College, Huazhong University of Science and Technology, Wuhan 430030, China; ^2^Bio-X Institutes, Key Laboratory for the Genetics of Developmental and Neuropsychiatric Disorders (Ministry of Education), Shanghai Jiaotong University, Shanghai 200240, China

## Abstract

**Purpose:**

Hydroxytyrosol (HT) processes multiaspect pharmacological properties such as antithrombosis and antidiabetes. The aim of this study was to explore the antistherosclerotic roles and relevant mechanisms of HT.

**Methods:**

Male apoE^−/−^ mice were randomly divided into 2 groups: the control group and the HT group (10 mg/kg/day orally). After 16 weeks, blood tissue, heart tissue, and liver tissue were obtained to detect the atherosclerotic lesions, histological analysis, lipid parameters, and inflammation. And the underlying molecular mechanisms of HT were also studied in vivo and in vitro.

**Results:**

HT administration significantly reduced the extent of atherosclerotic lesions in the aorta of apoE^−/−^ mice. We found that HT markedly lowered the levels of serum TG, TC, and LDL-C approximately by 17.4% (*p* = 0.004), 15.2% (*p* = 0.003), and 17.9% (*p* = 0.009), respectively, as well as hepatic TG and TC by 15.0% (*p* < 0.001) and 12.3% (*p* = 0.003), respectively, while inducing a 26.9% (*p* = 0.033) increase in serum HDL-C. Besides, HT improved hepatic steatosis and lipid deposition. Then, we discovered that HT could regulate the signal flow of AMPK/SREBP2 and increase the expression of ABCA1, apoAI, and SRBI. In addition, HT reduced the levels of serum CRP, TNF-*α*, IL-1*β*, and IL-6 approximately by 23.5% (*p* < 0.001), 27.8% (*p* < 0.001), 18.4% (*p* < 0.001), and 19.1% (*p* < 0.001), respectively, and induced a 1.4-fold increase in IL-10 level (*p* = 0.014). Further, we found that HT might regulate cholesterol metabolism via decreasing phosphorylation of p38, followed by activation of AMPK and inactivation of NF-*κ*B, which in turn triggered the blockade of SREBP2/PCSK9 and upregulation of LDLR, apoAI, and ABCA1, finally leading to a reduction of LDL-C and increase of HDL-C in the circulation.

**Conclusion:**

Our results provide the first evidence that HT displays antiatherosclerotic actions via mediating lipid metabolism-related pathways through regulating the activities of inflammatory signaling molecules.

## 1. Induction

It is well-recognized that atherosclerotic cardiovascular diseases (ASCVDs) including coronary artery disease and ischemic stroke are the “leading killer” threatening human life [1]. Epidemiological studies report that the number of patients with ASCVDs will continue to develop rapidly in the next decade, followed by a serious social-economic burden. Atherosclerosis, mainly occurring in large and middle elastic muscular arteries, is characterized by dynamic plaque injury driving to lumen stenosis and is a shared pathological process of ASCVDs [2].

An army of works illustrated that the dyslipidemia and inflammation are closely related to the burden and range of atheromatous plaque [3–6]. As a crucial organ for lipid metabolism, the liver plays an important role in the development of atherosclerosis [7]. For instance, blood LDL-C provides atheroprone effects and HDL-C possesses atheroprotective roles, respectively; both of which are regulated by liver cells [8–10]. Once the synthesis of LDL-C is enhanced or generation of HDL-C is inhibited in hepatocytes, the vascular wall has an increased risk of plaque development. Besides, a strong relationship between liver inflammation and atherosclerosis has been reported [10, 11]. Hepatocytes in patients with atherosclerosis inevitably suffer from mitochondrial dysfunction, immune status alteration, and necrosis, which induces liver inflammation, as seen by the overproduction of inflammatory factors including C-reactive protein (CRP), tumor necrosis factor- (TNF-) *α*, and leukocyte inflammatory chemical factors such as IL-6, IL-12, and monocyte chemoattractant protein-1 (MCP-1) [12]. There is evidence that inflammation, an important factor affecting intracellular lipid metabolism, exerts a nonnegligible effect on lipid metabolism disorders [13]. Furthermore, inflammatory responses are found to disturb the process of reverse cholesterol transport, implying that atherogenic roles of inflammation are partly ascribed to its regulation of lipid metabolism [14]. However, the underlying mechanisms how inflammation affects lipid metabolism are equivocal.

Hydroxytyrosol (HT) is a phenolic substance rich in virgin olive oil with excellent antioxidant, antimicrobial, and anticarcinogenic activities [15–17]. Recently, a considerable scientific attention is focused on the protective properties of HT against the cardiovascular diseases, diabetes, cancers, and macular degeneration [18, 19]. Results of Jemai et al. show that HT is capable of attenuating the lipid peroxidation in multiple organs and increasing antioxidant enzyme activities in the liver [20]. Besides, HT displays a lipid-lowering effect by suppressing the *de novo* synthesis of fatty acid and increasing the catabolism of fatty acid [18, 21]. Consequently, HT administration has been discovered to exhibit several antiatherosclerotic effects, including regulation of blood lipid profiles, and decrease of concentrations of inflammatory factors [22]. Thus, a dietary pattern with HT might be preferable for atherosclerosis patients. However, whether HT produces protective roles against atherosclerosis via regulating lipid metabolism through affecting inflammation in hepatocytes is poorly understood. According to these backgrounds, the aim of this study was to test the influence of HT administration in attenuating the progression of atherosclerotic lesions and liver steatosis and then discover relevant molecular mechanisms in vivo and in vitro.

## 2. Materials and Methods

### 2.1. Animal Experiment

All animal procedures in the present study strictly complied with the National Institutes of Health Guide for the Care and Use of Laboratory Animals under the approval by the Institutional Animal Care and Use Committee of Tongji Medical College, Huazhong University Science and Technology, Wuhan, China.

Eight-week-old apoE^−/−^ mice with the C57BL/6 genetic background purchased from Vital River Laboratory Animal Technology Co. Ltd. (Beijing, China) were acclimatized for 1 week before the experiment and then were ear-marked and randomly allocated into 2 groups (*n* = 11 per group) according to their weight. The control group and the HT group were fed with western-type diet (21% fat and 0.15% cholesterol). The HT (70604, Cayman Chemical, Michigan, USA) group was administrated additionally with HT at a dose of 10 mg/kg/day orally, and the control group was treated with the same volume of saline gavage. All animals were housed individually in the same room at a conditioned temperature of 22 ± 2°C under a standard 12 h light/dark cycle with food and water *ad libitum*. At the end of the 16^th^ week, all animals were fasted for 12 h and sacrificed, and then the blood samples, and heart tissues, aortic tissues, and liver tissues were harvested.

### 2.2. Analyses of Atherosclerotic Lesions

Lipid-rich atherosclerotic plaques in the entire aorta stained with Oil Red O (ORO, G1015, Servicebio Biotechnology, Wuhan, China) were quantified to analyze the extent of atherosclerosis. For en face analyses, the entire aorta from the proximal ascending aorta to the iliac artery bifurcation was dissected. Then, periadventitial tissues were rapidly cleaned and the aorta was opened longitudinally. After fixation in 4% paraformaldehyde solution overnight, the aorta was rinsed with PBS, incubated with 0.5% ORO for 2 h, destained in 70% ethanol, and washed with PBS. The aortic atherosclerotic plaque burden was assessed by the percentage of plaque area relative to the whole aorta area and was determined by Image-Pro Plus 6.0 software (Media Cybernetics, USA).

### 2.3. Histological and Morphometric Analyses

Tissues of the aorta and liver stored in 4% paraformaldehyde (AR1069, Boster Biological Technology, China) for 24 h were then dehydrated, embedded in paraffin, and subjected to cross sectioning. Sections (5 *μ*m) were obtained and stained with hematoxylin-eosin (HE, C0105, Beyotime Biotechnology, Beijing, China) and subsequently were observed with a microscope. The aorta section staining was performed for assessing the size of atherosclerosis lesions, and the liver section was used for observing the morphology changes.

The liver samples were embedded in optimum cutting temperature compound and frozen in the liquid nitrogen for few minutes and then were sliced by a cryostat. Cryosections of the liver (8 *μ*m) were stained in 0.5% ORO for observing the lipid accumulation in the liver visually.

### 2.4. Determination of Serum and Liver Lipid Parameters

Fasting serum samples were collected by retroorbital puncture and then stored at -80°C for detection of lipid profiles. Serum levels of TG, TC, LDL-C, and HDL-C were measured with the corresponding commercial kits (A110-1-1, A111-1-1, A113-1-1, and A112-1-1) according to the manufacture's recommendation (Jiancheng Bioengineering Institute, Nanjing, China). For TG and TC measurement, the samples and required agents in corresponding kits were mixed gently and incubated 10 minutes at 37°C. After that, the absorbance was examined at 510 nm. For LDL-C and HDL-C detection, the samples and required agents in kits were blended lightly, firstly incubated 5 minutes at 37°C, and the absorbance was examined at 546 nm, then mixed with another agent, incubated another 5 minutes at 37°C, and another absorbance was examined at 546 nm. We calculated the corresponding levels based on the absorbance values.

In order to discover the concentration of liver TG and TC, liver tissue with isopropyl alcohol was grounded thoroughly to 10% homogenate, and the lipid was sufficiently extracted overnight at 4°C. Then, the samples were centrifuged at 15000 rpm at 4°C for 30 minutes and the supernatant was obtained. Afterwards, the detection method and principle of supernatant TG and TC were the same as serum TG and TC.

### 2.5. Detection of Circulating Cytokines

Assessments for CRP (EK0977), TNF-*α* (EK0527), IL-1*β* (EK0394), IL-6 (EK0411), and IL-10 (EK0417) were performed as per quantitative ELISA kit protocols (Boster Biological Technology, China). Briefly, the samples and working agents in corresponding kits were processed in turn, and finally, the color reaction was terminated with the addition of stop solution, and immediately, the absorbance was assayed at 450 nm. The calculation method of the standard curve is based on the absorbance values, and the cytokine levels were examined.

### 2.6. Experiments with HepG2 Cells

Human hepatocarcinoma HepG2 cell line was obtained from American Type Culture Collection (ATCC, Manassas, VA, USA). The cells were routinely cultured in Dulbecco's modified Eagle's medium (DMEM, 11965-092, Gibco, Grand island, NY, USA) supplemented with 10% fetal bovine serum (FBS, 10270-106, Gibco, Grand island, NY, USA) and 1% penicillin-streptomycin (15140148, Gibco, Grand island, NY, USA) in a humidified incubator containing 5% CO_2_ and 95% air at 37°C. The cells were serially subcultured at 1 : 3 split ratio.

HT was prepared in dimethylsulfoxide (DMSO, D2650, Sigma-Aldrich, St. Louis, MO, USA) at a concentration of 100 mM. HepG2 cells were seeded in 6-well plates at a density of 5 × 10^5^ cells/well in 1 mL DMEM containing 10% FBS for 24 h for attachment. In the experiments with pharmacological inhibitor, the medium was replaced with fresh medium with solvent vehicle (DMSO) or diverse concentrations (10, 25, and 50 *μ*M) of HT or selective inhibitor of p38, SB203580 (SB, 20 *μ*M, S1076, Selleckchem, Houston, USA) for 12 h. An equal amount of DMSO, which the proportion is 1/1000, was always tested as the negative control to assure that the solvent vehicle has no influence.

### 2.7. Cell Viability Assay

The Cell Counting kit-8 (AR1199, Boster Biological Technology, China) was used to assess the cell viability following the manufacturer's protocol. HepG2 were seeded into 96-well plates at 0.7 × 10 [4]/well in DMEM with 10% FBS. Following this, the medium was refreshed after 4 h and solvent vehicle (DMSO) or diverse concentrations (10, 25, and 50 *μ*M) of HT or SB (20 *μ*M) were added into the DMEM. After the incubation period of 12 h, 10 *μ*L of CCK-8 solution was added into each well, and then, the plates were placed for another 2 h incubation at 37°C. The absorbance was examined at 450 nm.

### 2.8. Western Blot

The proteins of liver and cells were extracted by lysing with RIPA lysis buffer (Beyotime Institute of Biotechnology, China) with 1% protease inhibitor and protein phosphatase inhibitors. The concentrations of extracts were quantified using the BCA kit (AR1189, Boster Biological Technology, China). Subsequently, equal quantities of proteins (20 *μ*g) were separated using SDS-PAGE gel and transferred to polyvinylidene difluoride membrane filter (Merck Millipore, Darmstadt, Germany). After blocking with 5% BSA in TBST, the membrane was incubated overnight with respective primary antibodies at 4°C. Afterwards, the membranes were incubated with appropriate horseradish peroxidase-conjugated IgG for 1 h at room temperature. The protein bands were visualized using an enhanced ECL kit (AR1191, Boster Biological Technology, China) and were quantified by the ImageJ software (NIH, USA). These primary antibodies included ATP-binding membrane cassette transport protein (ABC) AI (1 : 1000, ab18180), SREBP2-M (1 : 1000, ab30682), LDL receptor (LDLR) (1 : 1000, ab52818), scavenger receptor B1 (SRBI) (1 : 1000, ab30682) (Abcam, Cambridge, UK), AMPK*α* (1 : 1000, #2532), P-AMPK*α* (1 : 1000, #2535), P38 (1 : 1000, #9212), P-p38 (1 : 1000, #9211), JNK (1 : 1000, #9252), P-JNK (1 : 1000, #4668), NF-*κ*B (1 : 1000, #8242), P-NF-*κ*B (1 : 1000, #3033), CRP (1 : 1000, #14316) (Cell Signaling Technology, Boston, USA); apoAI (1 : 1000, A14211), PCSK9 (1 : 1000, A7860) (ABclonal Biotech Co. Ltd., Cambridge, MA, USA), and IL-2 (1 : 1000, AF5105), IL-6 (1 : 1000, DF6087) (Affinity Bioscience, OH, USA). GAPDH (1 : 10000, AC002), and *β*-actin (1 : 10000, AC004) (ABclonal Biotech Co. Ltd., Cambridge, MA, USA) were blotted as control.

### 2.9. Statistical Analysis

Data was presented as the means ± SD for the indicated groups. SPSS version 22.0 software (IBM, Chicago, USA) was used for data analysis. The homogeneity of variances of data was tested by the Levene test. Student's unpaired *t*-test was used to analyze the differences between two groups. Statistical difference was considered significant at *p* < 0.05.

## 3. Results

### 3.1. HT Obviously Attenuated the Development of Atherosclerotic Lesions in apoE^−/−^ Mice

**Figure 1:**
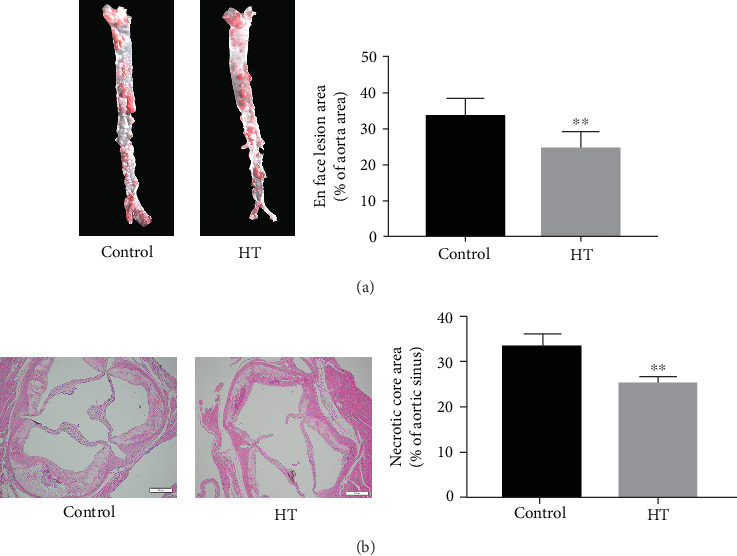
The effect analysis of HT on the atherosclerotic plaques of apoE^−/−^ mice. (a) Quantification of degree of aorta stenosis was detected by the lesion area/entire aorta area ratio via ORO staining. (b) The representative necrotic core area in cross-sections of the aortic sinus area was measured by HE staining. The data was presented as means ± SD, *n* = 6 for each group. ∗*p* < 0.05, ∗∗*p* < 0.01 vs. the control group.

At first, we tested the degree of atherosclerotic lesions in different groups via staining the entire aorta with ORO. After 16 weeks of HT administration, we found that HT treatment dramatically decreased the extent of atherosclerotic lesions in the en face of the total aorta to 24.61 ± 4.76% (*p* = 0.009) from 33.58 ± 4.76% in the control group (Figure 1(a)). On the other hand, results of HE-stained paraffin sections showed that oral administration of HT improved the development of plaque lesions, as seen by thinner intima, ampliative aortic lumen, shrinking necrotic core, and less subendothelial cholesterol crystals (Figure 1(b)).

### 3.2. HT Administration Improved the Circulating and Hepatic Lipid Spectrum and Alleviated the Liver Steatosis

**Figure 2:**
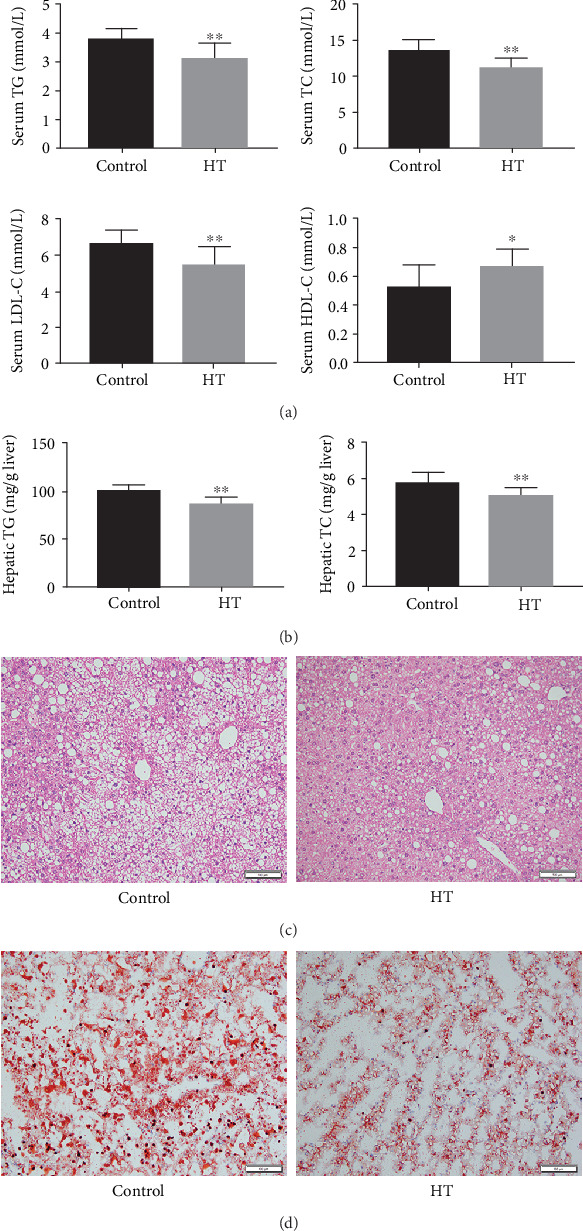
The effect of HT treatment on the lipid metabolisms of apoE^−/−^ mice. (a) The levels of serum lipid profiles. (b) The concentrations of hepatic lipid parameters. (c) Morphological alterations of liver cross-sections observed from mice in different groups. (d) Lipid accumulation in the liver surveyed by ORO-stained cryosections of each group. The data was present as means ± SD, *n* = 6 for each group. ∗*p* < 0.05, ∗∗*p* < 0.01 vs. the control group.

Then, we investigated the mechanisms by which HT extenuated the progression of atherosclerotic plaques. Given that dyslipidemia serves as a key pathological event responsible for atherogenesis, we firstly examined the levels of bloodstream and hepatic lipids. Our findings indicated that HT treatment significantly decreased the contents of serum TG, TC, and LDL-C and elevated the level of HDL-C compared to the control group approximately by 17.4% (*p* = 0.004), 15.2% (*p* = 0.003), 17.9% (*p* = 0.009), and 26.9% (*p* = 0.033), respectively (Figure 2(a)). As the liver is the central organ of lipid metabolism, we then measured the contents of hepatic TG and TC. A 15.0% decrease in hepatic TG (*p* < 0.001 versus control) and a 12.3% (*p* = 0.003 versus control) decrease in hepatic TC were observed in HT-treated mice (Figure 2(b)), which implied that HT was capable of attenuating the accumulation of lipids in the liver.

Afterwards, we investigated the effects of HT on the morphological characteristics of the liver and found that HE-stained liver sections of the HT group presented mild hepatic steatosis and slight morphological alterations when compared with the control group (Figure 2(c)). Similarly, we analyzed ORO-stained cryosections and observed that HT improved the lipid deposition in the hepatic tissue (Figure 2(d)).

### 3.3. HT Exerted Regulatory Effects in the Expression of Lipid Metabolism-Related Molecules and Inflammation-Related Factors in the Liver

**Figure 3:**
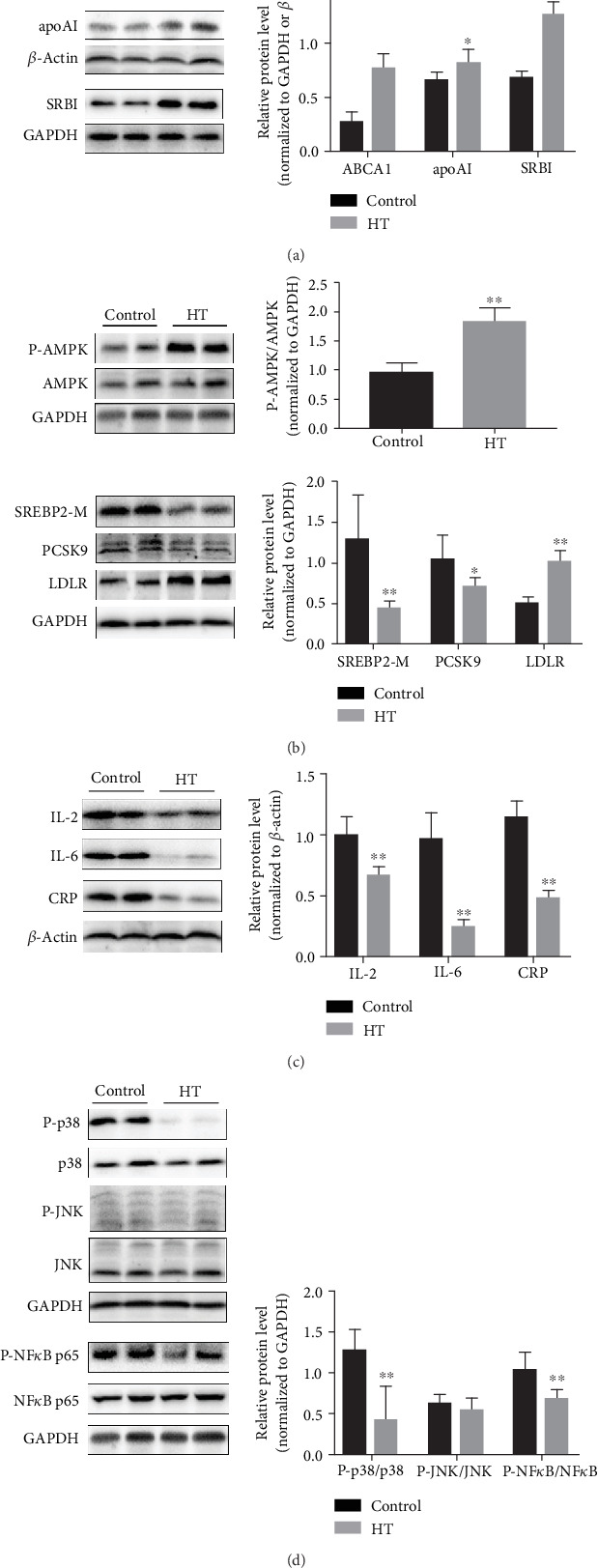
The protective roles of HT administration against atherosclerosis via the pathways of lipid turbulence and inflammation. (a) The expression of these molecules related to HDL synthesis and regulation, including ABCA1, apoAI, and SRBI. (b) The level of proteins responsible for LDL regulation. (c) The hepatic inflammatory factor expression and (d) its signal pathway molecules. The data was present as means ± SD, *n* = 6 for each group. ∗*p* < 0.05, ∗∗*p* < 0.01 vs. the control group.

Then, we investigated whether HT ameliorated lipid parameters through regulating relevant metabolic pathway molecules. Our data manifested that HT administration significantly elevated the expression of ABCA1 (*p* < 0.001) and SR-BI (*p* < 0.001) and induced a similar but less significant tendency of apoAI (*p* = 0.27) (Figure 3(a)). Moreover, it was reported that AMPK could affect the cholesterol level by inhibiting the activity of SREBP2, which further regulated downstream target genes including PCSK9 and LDLR in the liver. In this study, HT treatment increased the phosphorylated level of AMPK (*p* < 0.001) and reduced the contents of mature SREBP2 (SREBP2-M) (*p* = 0.004) and PCSK9 (*p* = 0.016) but elevated the expression of LDLR (*p* < 0.001) (Figure 3(b)). Given that the high inflammatory state of the liver accelerated the progression of atherosclerosis, the inflammatory biomarkers in the liver were examined. As showed in Figure 3(c), HT administration significantly lowered the expression levels of IL-2 (*p* = 0.001), IL-6 (*p* < 0.001), and CRP (*p* < 0.001) when compared to the control group. Then, we measured the inflammation-related signaling molecules and found that HT effectively weakened the activities of p38 MAPK (*p* = 0.002) and lowered the phosphorylated level of NF-*κ*B (*p* = 0.006), as seen in Figure 3(d). The results revealed that HT exerted regulatory effects on the signal pathways involved in lipid metabolism and inflammation in the liver.

### 3.4. HT Ameliorated the Systemic Inflammatory Status

**Figure 4:**
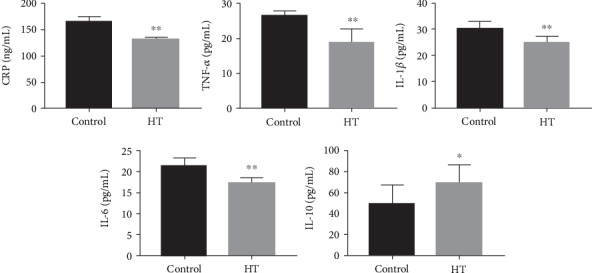
The serum inflammation-related parameters in animals subjected to HT or not, including proinflammatory factors CRP, TNF-*α*, IL-1*β*, IL-6, and anti-inflammatory factor IL-10. The data was present as means ± SD, *n* = 6 for each group. ∗*p* < 0.05, ∗∗*p* < 0.01 vs. the control group.

It was well-accepted that a multitude of inflammation-related cytokines participated in the initiation and progression of atherosclerosis. We then detected the inflammatory factors in vivo and observed that the levels of serum CRP, TNF-*α*, IL-1*β*, and IL-6 associated with proinflammatory status were reduced in the HT-treated mice approximately by 23.5% (*p* < 0.001), 27.8% (*p* < 0.001), 18.4% (*p* < 0.001), and 19.1% (*p* < 0.001), respectively. In the meantime, a 1.4-fold increase in anti-inflammatory factor IL-10 was found in the HT group compared to the control group (*p* = 0.014, Figure 4).

### 3.5. HT Alleviated the Lipid Disorders and Inflammation by Regulating the p38 Signal Pathway In Vitro

**Figure 5:**
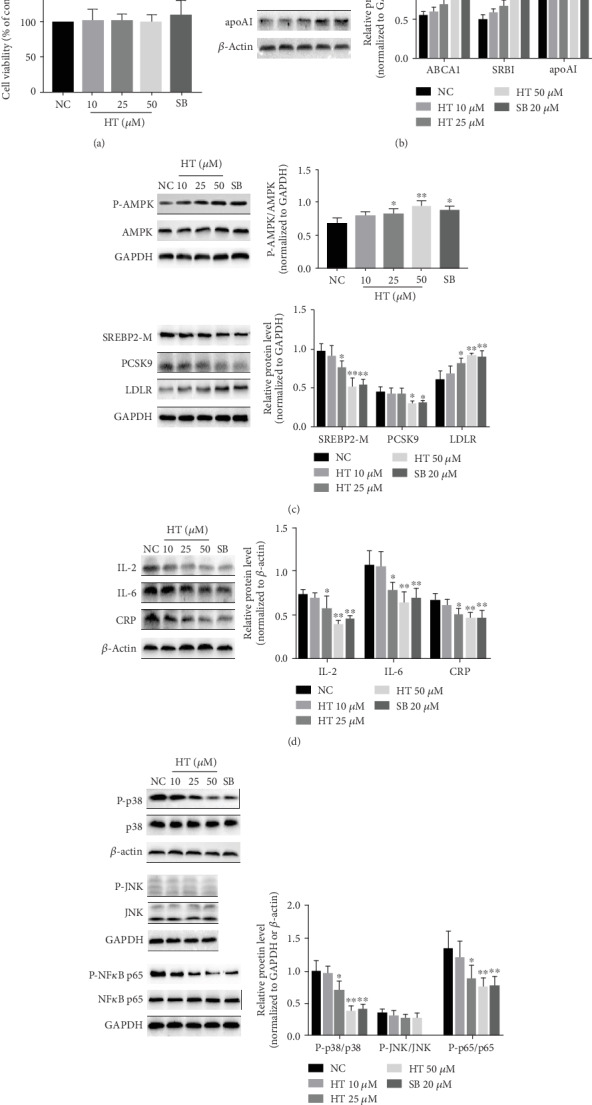
Effects of HT and selective inhibitor of p38 on lipid metabolism and inflammation pathway molecules. HepG2 cells treated with diverse concentrations (10, 25, and 50 *μ*M) of HT or SB203580 (20 *μ*M) for 12 h were subjected to CCK-8 assay and Western blot analysis. The data was present as means ± SD, *n* = 3 for each group. ∗*p* < 0.05, ∗∗*p* < 0.01 vs. the control condition. NC: negative control, namely, solvent vehicle control (DMSO). NS: not significant vs. DMSO. SB: SB203580.

Firstly, the effect of HT on the cellular viability of cultured HepG2 cells was detected. CCK8 assay showed that negative control and treated cells had no significant difference on the viability of HepG2 cells (Figure 5(a)). To further confirm the atherosclerotic protective mechanisms of HT, we designed the in vitro experiment. HepG2 cells were treated with DMSO or escalating concentrations of HT (10, 25, and 50 *μ*M) or SB203580 (20 *μ*M) for 12 h. As expected, HT caused a level increase of ABCA1 and apoAI in a dose-dependent manner, while the effect of HT with 10 *μ*M concentration was not significant. Besides, after cells were treated with HT, the phosphorylated level of AMPK was increased, and the expressions of mature SREBP2 (SREBP2-M) and PCSK9 were reduced, and the LDLR level was elevated at concentrations of 50 *μ*M. Moreover, the activities of the inflammation-related signal molecules including p38 and NF-*κ*B were decreased, accompanied with the weakened expression of IL-2, IL-6, and CRP (Figure 5).

At the same time, to further analyze whether HT regulated lipid metabolism and inflammation by regulating the p38 signal pathway, we examined the effects of selective inhibitor of p38 (SB203580) on the expression of these molecules above. Figure 5 shows that the inhibitor exhibited the similar actions with HT, as evidenced by the increased expression of P-AMPK (*p* = 0.012), LDLR (*p* = 0.002), ABCA1 (*p* < 0.001), and apoAI (*p* = 0.019) and decreased contents of SREBP2-M (*p* = 0.001), PCSK9 (*p* = 0.032), and P-NF-*κ*B (*p* = 0.009), followed by expression reduction of IL-2 (*p* = 0.002), IL-6 (*p* = 0.009), and CRP (*p* = 0.009). These findings suggested that the effective roles of HT in the regulation of lipid metabolism and inflammatory response were ascribed to the inhibition of the p38 signaling pathway, thereby leading to the downstream AMPK activation and the activity suppression of NF-*κ*B, respectively.

## 4. Discussion

It has been demonstrated that the initiation and progression of atherosclerosis is associated with a variety of pathological processes including subendothelial lipid accumulation and inflammation response [23]. Previous studies have clearly certificated a beneficial effect of HT against atherosclerosis [24], but the involved mechanisms are not fully understood [25]. Consistent with effects mentioned above, HT treatment obviously weakened the expansion of atheroma lesions and improved the serum and hepatic lipid profile as well as ameliorated hepatic steatosis and inflammatory status in apoE^−/−^ mice. Besides, we further discovered that the antiatherosclerotic mechanisms of HT were attributed to the improvement of lipid profile disorders via regulation of the inflammatory signal pathway. However, in contrast to these results, the report published by Acín et al. demonstrated the proatherosclerotic effects of the same concentration of HT for 10 weeks in apoE^−/−^ mice fed with a chow diet [26]. These differences in results may be due to the fact that a high-fat diet was used as feedstuff instead of a low-cholesterol diet, and the 16 weeks of treatment period instead of 10 weeks, and also study on high-cholesterol diet animal model found that HT improved the development of atherosclerosis and lipid disorders [27]. So further in vivo studies are required to confirm the precise effects of HT when taken in combination with different diet.

Multiple studies have confirmed that abnormal levels of blood lipids accelerate the pathological process of atherosclerosis. Thus, we evaluated the effects of HT on the lipid profiles and lipid metabolism-related signaling pathways in the apoE^−/−^ mice. By means of exporting cholesterols from peripheral tissues including the aorta and following transport towards the liver for elimination, HDL, a heterogeneous population of lipoprotein particles, serves as a key carrier in reverse cholesterol transport pathway, a kind of an antiatherogenic process in vivo [28, 29]. We found that HT gavage notably raised the concentration of circulating HDL-C, implying that there was a reduction in the cholesterol burden of the vascular wall, as evidenced by Figure 2. Furthermore, apoAI and ABCA1 displayed vital roles in processes of HDL biogenesis and early studies reported that loss-of-function mutations of these two genes could cause HDL deficiency [30], HT was likely to increase the level of HDL-C via inducing the expression of apoA-I and ABCA1 in hepatocytes, as seen by the results of Figures 3 and 5. Although epidemiological evidence has illuminated that the level of blood HDL-C is an inverse predictor of the incidence of atherosclerosis-related CVDs, several pharmacological approaches elevating HDL-C have failed to decrease the risk of diseases [31, 32], hinting that further investigations are needed for determining whether these lipoproteins are involved in antiatherosclerotic activities of HT, such as the detection of HDL functionality. In addition, SR-BI, an important mediator of HDL metabolism, mediates selective uptake of HDL cholesterol esters and participates in RCT [33]. Results in this work indicated that treatment with HT manifestly increased the expression of SR-BI, demonstrating the HT capacity facilitating the cyclic utilization of HDL.

**Figure 6:**
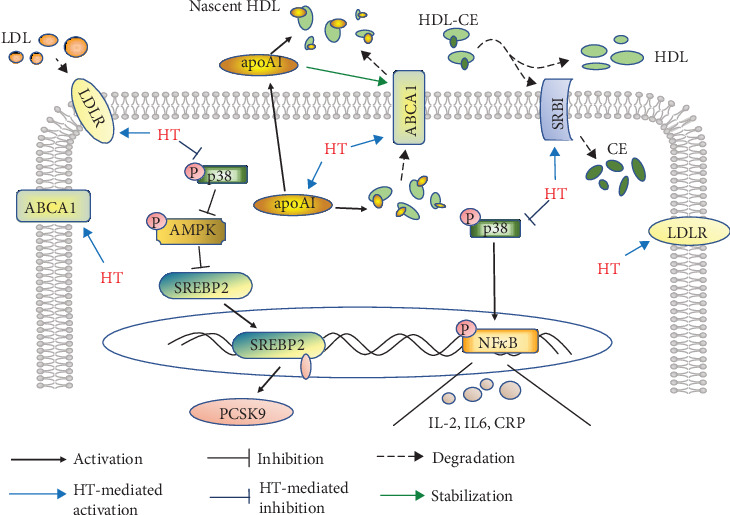
Schematic description of the protective mechanisms involved in the antiatherosclerosis actions of HT in apoE^−/−^ mice fed by western-type diet. CE: cholesterol ester.

Depending on the LDLR-mediated endocytosis, hepatocytes are responsible for clearing LDL-C particles in the circulation, leading to the decrease of the LDL-C level which is crucial for retarding the progression of atherosclerosis. SREPB2, an intracellular cholesterol sensor located in the endoplasmic reticulum, provides feedback regulation of intracellular cholesterol [34]. When the intracellular cholesterol level is low, inactive SREBP2 is cleaved by site 2 proteases in the Golgi to generate mature/active SREBP2 (SREBP2-M). Then, SREBP2-M migrates into the nucleus, where it initiates transcription of target genes, such as PCSK9 and LDLR. It is reported that PCSK9 triggers LDLR intracellular degradation by attaching to LDLR surface and translocating to lysosomes and the expression of PCSK9 positively related to the LDL-C level [35]. Our findings showed that HT treatment effectively lowered the level of serum LDL-C and reduced the expression of SREBP2-M and PCSK9 but conversely raised the level of LDLR in hepatocytes, which implied that HT facilitated the reduction of circulating LDL-C content possibly via inhibiting the SREBP2/PCSK9 pathway. But the LDLR increase was not in agreement with the tendency of SREBP2-M level change, and a possible reason accounting for this was that HT raised the LDLR expression by other signal pathways, which were needed to be further explored. Moreover, AMPK, a phylogenetically conserved serine/threonine protein kinase, is an energy sensor that controls intracellular energy metabolism [36]. Once activated, AMPK is capable of inducing a series of biological activities for regulating the lipid metabolism, including the suppression of SREBP2 activation and upregulation of PGC-1*α* [37]. In this study, the Western blot analysis indicated that HT administration obviously increased the activation of AMPK, suggesting that HT might activate AMPK to block the signal transduction of the downstream SREBP2/PCSK9 pathway, consequently resulting in the level reduction of blood LDL-C and alleviation of atherosclerosis development (Figure 6).

Cumulative evidences have indicated that inflammation facilitates the atherogenesis through triggering the disorders of lipid metabolism. For instance, inflammation promotes macrophage uptake of lipids and inhibits relevant lipid efflux [14]. In liver cells, inflammatory response could interrupt the expressions and activities of signaling molecules involved in lipid metabolism [38]. There is evidence that MAPKs and NF-*κ*B are the main inflammation-related molecules affecting the process of lipid metabolism [39]. It has been suggested that stress-activated p38 can expand lipid disorders by inhibiting the expression of LDLR through suppressing the p42/p44 MAPK pathway in hepatocytes [40]. Other studies demonstrate that activation of p38 potently disrupts the signal flow of the AMPK pathway. Furthermore, NF-*κ*B activation is discovered to significantly inhibit the expression of apoAI and ABCA1 and biogenesis of HDL [39, 41]. Here, our results showed that HT significantly repressed the activation of p38 and NF-*κ*B in hepatocytes, suggesting that the regulatory roles of HT in the lipid metabolism pathway might be partly ascribed to the activities suppression of p38 and NF-*κ*B. Afterwards, we performed in vitro experiments using HT intervention and p38 inhibitor SB203580 to further verify whether HT mediated lipid metabolism via regulating the p38 signal pathway. The data in this study showed that HT incubation significantly reduced the phosphorylation of p38, raised the level of phosphorylated AMPK and LDLR, and decreased the expression of SREBP2-M and PCSK9 in HepG2 cells. In addition, reduced content of phosphorylated NF-*κ*B and increased levels of apoAI and ABCA1 were also found in HT-treated cells. Besides, HT preincubation significantly altered the expression levels of HDL- and LDLR-related pathway molecules and inflammation-associated molecules in a concentration-dependent manner. Then, the use of SB203580 produced similar effects on these molecules above associated with inflammation response and lipid metabolism as compared to the administration of HT. These above findings suggested that HT might affect the concentrations of blood cholesterols via weakening the phosphorylation of p38, accompanied by activation of AMPK and inactivation of NF-*κ*B, which in turn triggered blockade of SREBP2/PCSK9 cascade and upregulation of LDLR, apoAI, and ABCA1, finally leading to reduction of LDL-C and increase of HDL-C in the circulation (Figure 6).

## 5. Conclusions

In conclusion, the present study shows that HT oral administration effectively decreases the extent of atheroma plaques in the aorta, regulates the profiles of blood lipid profiles, and alleviates the lipid deposition and inflammation in the liver of western-type diet-fed apoE^−/−^ mice. Our results provide the first evidence that HT displays antiatherosclerotic actions via mediating lipid metabolism-related pathways through regulating the activities of inflammatory signaling molecules.

## Data Availability

The data used to support the findings of this study are available from the corresponding author upon request.
